# UNDERSTANDING PROCESSES NEEDED TO SUCCESSFULLY IMPLEMENT PAL CARDS IN NURSING HOMES

**DOI:** 10.1093/geroni/igac059.444

**Published:** 2022-12-20

**Authors:** Megan Kelley, Miranda Kunkel, Reese Moore, Kamryn Kasler, Lexi Talmage, Alexandra Heppner, Kimberly Van Haitsma, Katherine Abbott

**Affiliations:** Miami University, Oxford, Ohio, United States; Miami University, Oxford, Ohio, United States; Miami University, Oxford, Ohio, United States; Miami University, Oxford, Ohio, United States; Miami University, Oxford, Ohio, United States; Miami University, Oxford, Ohio, United States; Pennsylvania State University, University Park, Pennsylvania, United States; Miami University, Oxford, Ohio, United States

## Abstract

**Background:**

The CFIR Process domain can be used to evaluate practices that are associated with successful or unsuccessful implementation. The purpose of this study was to understand the processes that led to successful implementation of the PAL Card QIP during the height of the pandemic from the provider champion’s perspective.

**Methods:**

Qualitative interviews with n=11 champions who completed the PAL Card QIP were audio recorded, transcribed verbatim, and coded using the CFIR Process Domain.

**Results:**

Major themes regarding the process of implementation included engaging (e.g., staff and family), executing (adapting to different situations), planning (e.g., having a clear plan of implementation), and reflecting and evaluating (e.g., taking time to evaluate the project). Discussion: Providers articulated strategies that contributed to successful implementation during a global pandemic, which can help inform future quality improvement projects. Implications for practice and policy will be discussed.

